# Health literacy education programmes developed for qualified health professionals: a scoping review protocol

**DOI:** 10.12688/hrbopenres.13386.1

**Published:** 2021-09-02

**Authors:** Lauren Connell, Yvonne Finn, Rosie Dunne, Jane Sixsmith

**Affiliations:** 1 Discipline of Health Promotion, National University of Ireland, Galway, Galway, Ireland; 2 CDA Diabetic Foot Disease: from PRevention to Improved Patient Outcomes (CDA DFD PRIMO) programme, National University of Ireland, Galway, Galway, Ireland; 3 Alliance for Research and Innovation in Wounds (ARIW), National University of Ireland, Galway, Galway, Ireland; 4 School of Medicine, National University of Ireland, Galway, Galway, Ireland; 5 James Hardiman Library, National University of Ireland, Galway, Galway, Ireland

**Keywords:** health literacy, health professional education, communication skills

## Abstract

**Introduction:** Health professional education for health literacy has been identified as having the potential to improve patient outcomes and has been recognized as such in policy developments. Health literacy is an emerging concept encompassing individuals’ skills and how health information is processed in relation to the demands and complexities of the surrounding environment. Focus has been predominantly on the dimension of functional health literacy (reading, writing and numeracy), although increasing emphasis has been placed on interactive and critical domains. Such dimensions can guide the development of health professional education programmes and bridge the gap in the interaction between health professionals and their patients. Currently little is known about qualified health professional’s education for health literacy, its development, implementation or evaluation.

**Aim:** To identify and map current educational interventions to improve health literacy competencies and communication skills of qualified health professionals.

**Methods: **A scoping review will be conducted drawing on methods and guidance from the Joanna Briggs Institute, and will be reported according to the Preferred Reporting Items for Systematic Review and Meta-Analyses extension for Scoping Reviews (PRISMA-ScR) Checklist. This study will retrieve literature on health professional education for health literacy through a comprehensive search strategy in the following databases: CINAHL; Medline (Ovid); the Cochrane Library; EMBASE; ERIC; UpToDate; PsycINFO and Central Register of Controlled Trials (CENTRAL). Grey literature will be searched within the references of identified articles: Lenus; ProQuest E-Thesis Portal; the HSE health research repository and RIAN. A data charting form will be developed with categories agreed by the research team, including: article details, demographics, intervention details, implementation and evaluation methods.

**Conclusion:** Little is known about the extent and nature of the current evidence base therefore in order to identify programmes and consolidate their demographics and characteristics within health literacy competencies and communication skills, a scoping review is warranted.

## Introduction

The need for health professional education in health literacy (HL) to improve patient outcomes has been identified ^1^, is supported by research literature ^1– 3^ and is recognised in policy development in European countries ^4^. HL is a public health issue and evolving concept that describes the personal skills and environment that enables individuals to obtain, understand and utilise information to make decisions that impact health status ^5^. Skills pertaining to adequate health literacy are inherently individual and dependent on the individuals’ socioeconomic environment ^6, 7^.

HL is defined by three core domains: functional, interactive and critical ^5^. At an individual level, functional HL leads to improved awareness of health risks, health services and treatment adherence; interactive HL leads to improved independence, motivation and self-confidence; whereas critical HL leads to better resilience to antecedents such as social adversity ^8^. The majority of the literature focuses on functional HL, however, there has been increasing emphasis on the development of the interactive dimension of HL. This has been particularly evident within health professional education, where programmes have been developed to improve HL competencies and communication skills ^9, 10^. Although often recognized as a separate entity ^11^, communication plays a significant role in the development of interactive and critical HL, whereby effective communication maintains the patient-practitioner relationship ^12, 13^. This communication takes place within the ‘oral exchange’ between the patient and professional, therefore recognizing the role of oral communication within HL and enhancing patient-practitioner interaction ^13^.

HL has been linked to health status and health service utilization, as higher HL levels have been found to be positively related to self-rated health status, disease knowledge, preventative care, and perceived health status; while being negatively correlated with hospitalization and emergency department visits ^14, 15^. In the European Health Literacy Survey (2009-2012), it was found that almost half of all adults studied had inadequate or limited HL skills which negatively impacts on their health ^16^. For people with chronic disease, limited HL has been associated with lower health-related quality of life (HRQoL) ^17^, and poorer health outcomes ^18^.

In Ireland, it is estimated that the major chronic diseases (cardiovascular disease, respiratory disease and diabetes) will increase by 20%–30% in the next five years ^19^. Diabetes has a profound effect on individuals with varying complications: macrovascular complications such as cardiovascular disease, stroke, peripheral vascular disease; and microvascular complications such as nephropathy, retinopathy, peripheral neuropathy, and diabetic foot disease ^20^. In diabetes, it has been found that improved patient-practitioner communication has the ability to improve patient diabetes behaviour, self-care and diabetes specific outcomes ^21^. Such self-care behaviours have been suggested to be linked to health literacy, where higher HL levels result in better self-care behaviours ^22, 23^. Interactive and critical HL have been found to be more influential than functional HL in influencing self-efficacy in those with diabetes ^24– 26^. In contrast, some studies have not found HL to have a statistically significant relationship with diabetes-related health outcomes such as wound healing ^18^ and other complications ^27^. However, in the aforementioned studies it must be noted that functional HL was assessed in each patient sample and does not portray how interactive and critical HL domains may effect patient health outcomes. A systematic review with meta-analysis found that overall, health-literacy-sensitive diabetes management interventions were effective in reducing HbA1C levels ^28^ The need for health professionals to implement communication strategies in practice with people with limited health literacy in order to develop their capacity for self-management was identified. Patient self-management has been considered in relation to the critical health literacy domain ^10^. For this identified need to be addressed qualified health professionals require health literacy competencies and communication skills. 

HL research has developed and grown since at least 1973 ^29^, however limited research has been undertaken on HL interventions and their effectiveness ^18^, particularly in regards to health professional education, despite the identification of such education programmes being relevant to mitigating potential health outcomes ^1^. More recently, some training programmes have been developed to address HL competencies and communication skills mainly for health professional students ^10, 30, 31^. Nevertheless, the extent and nature of programmes, needs identifying and collating to assess the potential of undertaking a full systematic review ^32^ and to inform future development of these complex interventions. Current educational health literacy interventions aimed at qualified health professionals need to be identified accordingly to collate the current evidence base and provide a comprehensive narrative pertaining to the characteristics, including their generic or any disease specific focus, methodologies and assessments used. This protocol is for a scoping review which aims to identify and map current educational interventions to improve Health Literacy competencies and communication skills of qualified health professionals.

## Methods

The extent and nature of research in relation to health literacy education programmes for qualified health professions is currently unknown. A preliminary review of research identified limited literature in the area. As a consequence, a scoping review design is appropriate to develop an overview of what is known ^33^ and to assess if a systematic review is possible ^34^. This scoping review will be conducted drawing on methods and guidance from the Joanna Briggs Institute ^35^, which adds to earlier guidance on scoping review methodology ^32^. It will be reported according to the Preferred Reporting Items for Systematic Review and Meta-Analyses extension for Scoping Reviews (PRISMA-ScR) Checklist ^34^.

Protocol development started with preliminary research which did not identify current literature within the population pertaining to those with either diabetic foot disease (DFD) or those with a diabetes diagnosis, therefore it was decided to expand the review to capture all qualified health professionals practicing in all settings.

The “PCC” mnemonic was used to formulate the review title, where PCC stands for Population, Concept and Context ^35^. The PCC mnemonic helps construct a title without the need for outcomes, interventions or phenomena of interest, like within a systematic review, however it may include elements of each. In this scoping review the population is qualified health professionals of all backgrounds. Concept refers to education programmes for health literacy competencies and communication skills. The context is in terms of qualified health professionals working in a clinical setting.

Five stages of a six stage framework will be used to structure this review ^32^, the optional stage six which comprises stakeholder consultation will not be adopted in the context of this stage of this current study.

### Stage 1: Identifying the research question

The primary research question is:

1. What health literacy competencies and communication skills educational interventions exist for qualified health professionals?

The secondary research questions are:

1. Of the qualified health professional education interventions identified which are focused on diabetes care?
2. What health literacy competencies and communication skills are integrated into each programme?
3. What are the characteristics of each education programme?
4. What were the barriers and facilitators to implementation?
5. What methods are used to evaluate intervention effectiveness? If any.
6. What are the outcomes of the education programme on qualified professionals and/or patients?

### Stage 2: Identifying relevant studies

This study will retrieve evidence through a comprehensive search strategy ( Table 1) in the following databases: CINAHL; Medline (Ovid); the Cochrane Library; EMBASE; ERIC; UpToDate; PsycINFO and Central Register of Controlled Trials (CENTRAL).

**Table 1.** Search Strategy for Medline (Ovid).

**1**	(("healthcare" or "health care") adj2 (professional* or provider* or personnel or worker*)).tw. or health personnel/
**2**	exp education/
**3**	(education adj2 (continuing or "competency based" or "competency-based" or health or program or programme*)).tw.
**4**	(workshop* or (problem-based adj (curricul* or learning))).tw. or ("problem based" adj2 (curricul* or learning)).mp. or (learning adj2 (active or experiential or problem-based or "problem based or case-based" or "case based")).tw.
**5**	(training adj2 (course* or module* or program or programme*)).tw.
**6**	training.tw. or inservice training/ or intervention*.tw. or course*.tw. or module*.tw.
**7**	staff development/ or clinical competence/ or program evaluation/ or program development/ or continu* professional development.tw.
**8**	2 or 3 or 4 or 5 or 6 or 7
**9**	exp Health Literacy/ or "health literacy".mp. or exp "health promotion"/ or "health literacy education".tw.
**10**	("health literacy" or ("health literacy" adj2 (competenc* or skill* or knowledge or attitudes))).tw.
**11**	communication skill*.tw.
**12**	(communication* adj2 ("teach back" or "teach-back" or method* or personal or program or social or personnel or health or nonverbal or non-verbal)).tw.
**13**	(skill* adj2 (interpersonal or social)).tw.
**14**	9 or 10 or 11 or 12 or 13
**15**	1 and 8 and 14
**16**	limit 15 to (english language and yr="1973 - 2021")

Grey literature will be searched within the references of identified articles; Lenus; ProQuest E-Thesis Portal; the HSE health research repository and RIAN. The search strategy was populated from a combination of free text search terms, text words, Medical Subject Headings (MeSH) terms and keywords with Boolean operators. Search terms will be used in combination with search filters to tailor for each database. The search was developed with advice from a research librarian with expertise in the area of strategy development. The selected keywords and search string, relevant to Medline via Ovid, can be found in Table 1 below.

Results from the search will be imported into Rayyan ^36^, a scoping review manager software, whereby citations will be collated and duplicates will be removed. Although no current studies exist regarding the reliability and efficacy of using such automation tools, users have noted that the use of these tools saved time and increased accuracy ^37^.

### Stage 3: Study selection

The search will be limited to the English language due to the variation in interpretations of the notion of HL from a cultural and socioeconomic perspective ^6, 7^. All searches will be limited to post- 1973, due to the history of HL research emerging at this time ^29^. Intervention components must contain health literacy competencies or communication skills training in order to be included, due to the interpretative nature of HL, the third author will be consulted if any discrepancies in interpretation arise. In this current study, health professionals identified will not be limited by profession or the setting in which they work. Study selection will be guided based on the following inclusion criteria:

- Population: Qualified health professionals.
- Settings: All settings.
- Intervention: HL competencies and communication skills education.
- Study Methods: All research methodologies.
- Limited to 1973-2021; adult patient populations (>18 years old).

And exclusion criteria:

- Population: Healthcare students.
- Literature pre- 1973.
- Paediatric patient populations (<18 years old).
- Not in the English language.

Exclusion criteria are based on not meeting all of the required inclusion criteria. Similar to previous research, the selection of sources and evidence will take place over four steps ^38^:

Step 1: Initial retrieval of sources, which will be performed by one author.

Step 2: Title screening. Titles will be screened against the inclusion criteria and will be retained if they explicitly meet the inclusion criteria. This step will be performed by two blinded authors, whereby the third author will mediate if any disagreements arise.

Step 3: Abstract screening. Abstracts will be screened against the inclusion criteria and will be retained if they meet the inclusion criteria. This step will be performed by two blinded authors. Disagreements will be mediated by the third author through discussion.

Step 4: Full text review. Articles will be retained if compliant with inclusion criteria. This will be performed by two authors of the research team and cross-checked with the third if any complications arise. Numbers of articles included and excluded will be documented using the PRISMA-ScR standardised template ^34^.

Prior to proceeding to Stage 4: “Charting the data”, a pilot sample of ten articles will be extracted by two authors, as a form of pilot testing, to ensure methods are reproducible and to allow extraction form revision if needed. On completion, this will allow the team to proceed to Stage 4.

### Stage 4: Charting the data

The extraction form will be collated based on the JBI template source of evidence details, characteristics and results extraction instrument ^35^, training programme evaluation methods ^39^ and insight from previous work ^40^. A data charting form will be developed drawing on categories, as agreed by the research team, such as: article details, demographics, intervention details, implementation and evaluation methods. An excel spreadsheet will be used to chart the data.

### Stage 5: Collating, summarizing, and reporting of results

Data will be reported for each selected study within each category as agreed on in the previous stage. Findings will be presented in a table that outlines the research demographics as defined in Stage 4. Any subcategories of emerging themes will be identified depending on presenting data. Entries will be checked by all authors.

### Dissemination

The findings of this scoping review will be published in a peer-reviewed journal and made available on ARAN, an NUI Galway open access repository, subject to the open-access policies of the original publishers.

### Study status

Not yet initiated.

## Conclusions

Although some training programmes have been developed to address HL competencies and communication skills mainly for health professional students ^10, 30, 31^, the extent and nature of programmes, needs identifying and collating to assess the potential of undertaking a full systematic review ^32^. This will inform future development of these complex interventions. Current educational health literacy interventions aimed at qualified health professionals need to be identified accordingly to collate the current evidence base and provide a comprehensive narrative pertaining to the characteristics, including their generic or any disease specific focus, methodologies and assessments used. This protocol is for a scoping review which aims to identify and map current educational interventions to improve health literacy competencies and communication skills of qualified health professionals, and to identify interventions within diabetes care. Little is known about the extent and nature of the current evidence base, particularly within diabetes care, therefore in order to identify programmes and consolidate their demographics and characteristics within health literacy competencies and communication skills, a scoping review is warranted.

## Data availability

No data are associated with this article.
