# Health literacy education programmes developed for qualified health professionals: a scoping review protocol

**DOI:** 10.12688/hrbopenres.13386.2

**Published:** 2022-01-11

**Authors:** Lauren Connell, Yvonne Finn, Rosie Dunne, Jane Sixsmith

**Affiliations:** 1 Discipline of Health Promotion, National University of Ireland, Galway, Galway, Ireland; 2 CDA Diabetic Foot Disease: from PRevention to Improved Patient Outcomes (CDA DFD PRIMO) programme, National University of Ireland, Galway, Galway, Ireland; 3 Alliance for Research and Innovation in Wounds (ARIW), National University of Ireland, Galway, Galway, Ireland; 4 School of Medicine, National University of Ireland, Galway, Galway, Ireland; 5 James Hardiman Library, National University of Ireland, Galway, Galway, Ireland

**Keywords:** health literacy, health professional education, communication skills

## Abstract

**Introduction:** Health literacy education, for health professionals, has been identified as having the potential to improve patient outcomes and has been recognized as such in policy developments. Health literacy, as a relational concept, encompasses individuals’ skills and how health information is processed in relation to the demands and complexities of the surrounding environment. Focus has been predominantly on the dimension of functional health literacy (reading, writing and numeracy), although increasing emphasis has been placed on interactive and critical domains. Such dimensions often guide the development of health professional education programmes, where the aim is to enhance the patient-practitioner relationship, and ultimately reduce the health literacy burden experienced by patients navigating health services. Currently little is known about qualified health professionals’ education in health literacy and communication skills, and development, implementation or evaluation of such interventions.

**Aim:** To identify and map current educational interventions to improve health literacy competencies and communication skills of qualified health professionals.

**Methods**: A scoping review will be conducted drawing on methods and guidance from the Joanna Briggs Institute, and will be reported according to the Preferred Reporting Items for Systematic Review and Meta-Analyses extension for Scoping Reviews (PRISMA-ScR) Checklist. This study will retrieve literature on health professional education for health literacy and communication skills through a comprehensive search strategy in the following databases: CINAHL; Medline (Ovid); the Cochrane Library; EMBASE; ERIC; UpToDate; PsycINFO. Grey literature will be searched within the references of identified articles; Lenus; ProQuest E-Thesis Portal; RIAN and OpenGrey. A data charting form will be developed with categories including: article details, demographics, intervention details, implementation and evaluation methods.

**Conclusion:** Little is known about the extent and nature of the current evidence base therefore a scoping review will be conducted, in order to identify programme characteristics in relation to health literacy competencies and communication skills.

## Introduction

The need for health literacy (HL) education, for qualified health professionals (QHPs), to improve patient outcomes has been identified ^1^, is supported by research literature ^1– 3^ and is recognised in policy development in European countries ^4^. This protocol is for a scoping review which aims to identify and map current educational interventions to improve HL competencies and communication skills of QHPs. Focus will be applied to diabetes care, as this study is a component of a larger research project entitled, Diabetic Foot Disease: from PRevention to treatment to IMproved patient Outcomes ( *DFD PRIMO*).

HL has been described as an ‘evolving’ concept ^5^, developing over time with multiple definitions identified in the literature ^6, 7^. This is an identified limitation to research and can negatively impact the measurement of HL ^8^. Nevertheless, there is increasing consistency in the use of a typology of HL comprising of three core domains: functional, communicative/interactive and critical ^5^. At an individual level, functional HL leads to improved awareness of health risks, health services and treatment adherence; interactive HL, also referred to as communicative HL, leads to improved independence, motivation and self-confidence; whereas critical HL leads to better resilience to antecedents such as social adversity ^9^.

A relational concept of HL will be used ^10^, focusing on an organisational health literacy (OHL) approach which makes health services easier for patients and their families to access, navigate and engage with so that they can make informed decisions and take informed actions for their health ^11^. In this conceptualisation, emphasis is not on the individuals’ capabilities to manage their own health but on how their environment and the health services can play a central role in their successful application of their abilities to access and utilise services. This approach is informed by the identification of the ten attributes of a HL friendly organisation ^12^, specifically that the organisation ‘uses health literacy strategies in interpersonal communications and confirms understanding at all points of contact’. By adopting this approach, educating QHPs on HL competencies, to optimise patient-practitioner communication ^13, 14^, has the potential to strengthen the patient-healthcare professional dyad. Such competencies include the knowledge, attitudes and skills that professionals need to master in order to appropriately address limited HL levels presenting in their patients ^15^. As a result health professional education in HL is often directed towards improving HL related communication skills by utilising a range of techniques such as teach-back ^16^, minimising jargon ^17^, Ask Me Three, which helps confirm patient understanding ^11^, and designing health literate reading materials to improve comprehensibility ^17^.

For the purpose of this research, the relational characteristic of HL is recognised and informs the choice of definition used which is that HL is ‘People’s ability to find, understand, appraise and communicate information to engage with the demands of different health contexts to promote health across the lifecourse’ ^10^.

In Ireland, 1 in 7 adults have been found to have limited HL skills ^18^, and at a European level almost every second respondent within the European health literacy survey (HLS-EU) had limited HL ^19^, which is associated with increased hospitalization, higher all-cause mortality, poor health related knowledge, self-care behaviour and other outcomes ^20^. A social gradient can be seen with a higher proportion of those with limited HL experiencing lower socio-economic status, lower educational attendance and attainment, and are of older age which mirrors the pattern of inequality of those with chronic diseases ^21, 22^.

For people with chronic disease, limited HL has been associated with lower health-related quality of life (HRQoL) ^23^, and poorer health outcomes ^24^. In chronic disease such as diabetes, demands on individuals are characterised by a high level of complexity ^25^, where self-management relies on patients’ having advanced HL skills, in order to utilise written education material and verbal instructions ^26^. Diabetes has a profound effect on individuals with varying complications: macrovascular complications such as cardiovascular disease, stroke, peripheral vascular disease; and microvascular complications such as nephropathy, retinopathy, peripheral neuropathy, and diabetic foot disease ^27^.

Inadequate HL has been shown to be an independent predictor of poor glycaemic control, being associated with a lower likelihood of achieving tight control ^28^. Also, it is suggested that when HL is considered in isolation it is associated with greater diabetes self-efficacy ^29– 31^, where greater self-efficacy is associated with lower glycaemic levels. It is implied that a positive relationship between HL and improved diabetes control. Interactive and critical HL have been found to be more influential than functional HL in influencing self-efficacy in those with diabetes ^32– 34^. In contrast, some studies have not found HL to have a statistically significant relationship with diabetes-related health outcomes such as wound healing ^24^ and other complications ^35^. But, when interactive HL or critical HL are considered some relationships have been found to be positive ^32, 33, 36^.

The majority of the literature focuses on functional HL, however, there has been increasing emphasis on the development of the interactive dimension of HL. This has been particularly evident within health professional education, where programmes have been developed to improve HL competencies and HL related communication skills ^15, 37^. If the HL demand placed on individuals is reduced, by means of improved communication and health literate communication from the QHPs, patient outcomes have the potential to improve ^38^. Limited evidence has shown that confirming patient’s understanding of new concepts can increase glycaemic control in those with diabetes ^39^.

Although HL research has developed and grown since at least 1973 ^40^, limited research has been undertaken on HL interventions and their effectiveness ^17^, particularly in regards to qualified health professional education, despite the identification of such education programmes being relevant to mitigating potential health outcomes ^1^. More recently, some training programmes have been developed, for QHPs, to address HL competencies and Hl related communication skills ^2, 37, 41, 42^. Although there is emerging evidence of these interventions, the extent and nature of programmes need to be collated in order to assess the potential of undertaking a full systematic review ^43^ and to inform future development of these complex interventions.

A HL education programme consists of a set of competencies to be addressed and achieved. Such competencies include the knowledge, attitudes and skills that professionals need to master in order to appropriately address limited HL levels presenting in their patients ^15^. Although often recognized as a separate entity ^10^, communication plays a significant role in the development of interactive and critical HL, whereby effective communication maintains the patient-practitioner relationship ^13, 14^.

Interactive HL has been found to be the most important HL domain needed within diabetes self-management ^44^, where interactive HL consists of a higher level of communication (oral literacy) and socials skills needed to extract and discuss information with others ^5^. Patients with these skills are characterized by the self-confidence to act independently on advice, and to interact effectively with the health system. Interactive/communicative HL takes place within the ‘oral exchange’ in the QHP and patient interaction ^14, 45^. Oral literacy and social skills are integral to the interactive HL domain and in meeting patients’ health needs and understanding. An ‘interactive communication loop’ has been recommended, whereby the QHP assesses patient understanding and recall ^39^; an example of this is the ‘Teach-Back’ tool ^16^. Other forms of communication within a health literate organisation include communicating: using social media and other digital forms, at an interprofessional level, with external stakeholders and at a community level.

Current educational health literacy interventions aimed at qualified health professionals need to be identified accordingly to collate the current evidence base and provide a comprehensive narrative pertaining to the characteristics, including their generic or any disease specific focus, methodologies and assessments used. Currently, there are no universally accepted guidelines in relation to development of HL curricula for qualified health professionals, although there are general outlines to help guide development such as the Calgary Charter on Health Literacy ^46^. Established HL competencies have been defined more recently for health professionals in areas such as general HL knowledge; HL related communication skills; and attitudes in practice ^47, 48^.

## Methods

The extent and nature of research in relation to health literacy education programmes for qualified health professions is currently unknown. A configurative scoping review was chosen as it aims to ‘seek concepts to provide enlightenment through new ways of understanding’ ^49^. A preliminary review of research identified limited literature in the area. As a consequence, a scoping review design is appropriate to develop an overview of what is known ^50^ and to assess if a systematic review is possible ^34^. An iterative approach will be used in this study to allow authors to develop the inclusion and exclusion criteria while considering the presenting evidence ^49, 51^. This scoping review will be conducted drawing on methods and guidance from the Joanna Briggs Institute ^52^, which adds to earlier guidance on scoping review methodology ^31^. It will be reported according to the Preferred Reporting Items for Systematic Review and Meta-Analyses extension for Scoping Reviews (PRISMA-ScR) Checklist ^53^. Protocol development started with preliminary research which did not identify current literature within the population pertaining to those with either diabetic foot disease (DFD) or those with a diabetes diagnosis, therefore it was decided to expand the review to capture all qualified health professionals (QHPs) practicing in primary, secondary and tertiary care settings.

The “PCC” framework was employed in this scoping review to determine the research question, whilst drawing on methods from Joanna Briggs Institute ^52^ and Arksey and O’Malley's (2005) scoping review framework ^43^. The PCC framework, where PCC stands for Population, Concept and Context ^52^, helps construct a title without the need for outcomes, interventions or phenomena of interest ^52^. The PCC framework provides the core detail on the inclusion criteria related to the review topic, but acknowledges the need for more detail when planning the screening phases. In this scoping review the population is qualified health professionals of all backgrounds. Concept refers to education programmes for health literacy competencies and health literacy related communication skills. The context is in terms of qualified health professionals working clinically in primary, secondary and tertiary care settings.

Five stages of a six stage framework will be used to structure this review ^43^, the optional stage six which comprises stakeholder consultation will not be adopted in the context of this stage of this current study. Nevertheless, this research is the first stage of a three stage project with the results of this scoping review informing stakeholder engagement activities and further research.

### Stage 1: Identifying the research question

The primary research question is:

1. What health literacy competencies and health literacy related communication skills educational interventions exist for qualified health professionals?

The secondary research questions are:

1. Of the qualified health professional education interventions identified which are focused on diabetes care?
2. What health literacy competencies and health literacy related communication skills are integrated into each programme?
3. What are the characteristics of each education programme?
4. What were the barriers and facilitators to implementation?
5. What methods are used to evaluate intervention effectiveness? If any.
6. What are the outcomes of the education programme on qualified professionals and/or patients?

### Stage 2: Identifying relevant studies

This study will retrieve evidence through a comprehensive search strategy ( Table 1) in the following databases: CINAHL; Medline (Ovid); the Cochrane Library; EMBASE; ERIC; UpToDate; PsycINFO.

**Table 1.** Search Strategy for Medline (Ovid).

**1**	(("healthcare" or "health care") adj2 (professional* or provider* or personnel or worker*)).tw. or health personnel/
**2**	exp education/
**3**	(education adj2 (continuing or "competency based" or "competency-based" or health or program or programme*)).tw.
**4**	(workshop* or (problem-based adj (curricul* or learning))).tw. or ("problem based" adj2 (curricul* or learning)).mp. or (learning adj2 (active or experiential or problem-based or "problem based or case-based" or "case based")).tw.
**5**	(training adj2 (course* or module* or program or programme*)).tw.
**6**	training.tw. or inservice training/ or intervention*.tw. or course*.tw. or module*.tw.
**7**	staff development/ or clinical competence/ or program evaluation/ or program development/ or continu* professional development.tw.
**8**	2 or 3 or 4 or 5 or 6 or 7
**9**	exp Health Literacy/ or "health literacy".mp. or exp "health promotion"/ or "health literacy education".tw.
**10**	("health literacy" or ("health literacy" adj2 (competenc* or skill* or knowledge or attitudes))).tw.
**11**	communication skill*.tw.
**12**	(communication* adj2 ("teach back" or "teach-back" or method* or personal or program or social or personnel or health or nonverbal or non-verbal)).tw.
**13**	(skill* adj2 (interpersonal or social)).tw.
**14**	9 or 10 or 11 or 12 or 13
**15**	1 and 8 and 14
**16**	limit 15 to (english language and yr="1973 – 2021")

Grey literature will be searched within the references of identified articles; Lenus; ProQuest E-Thesis Portal; RIAN and OpenGrey. The search strategy was populated from a combination of free text search terms, text words, Medical Subject Headings (MeSH) terms and keywords with Boolean operators. Search terms will be used in combination with search filters to tailor for each database. The search was developed with advice from a research librarian with expertise in the area of strategy development. The selected keywords and search string, relevant to Medline via Ovid, can be found in Table 1 below.

Results from the search will be imported into Rayyan ^54^, a scoping review manager software, whereby citations will be collated and duplicates will be removed. Although no current studies exist regarding the reliability and efficacy of using such automation tools, users have noted that the use of these tools saved time and increased accuracy ^55^.

### Stage 3: Study selection

The search will be limited to the English language due to the variation in interpretations of the notion of HL from a cultural and socioeconomic perspective ^56, 57^. All searches will be limited to post- 1973, due to the history of HL research emerging at this time ^40^. Intervention components must contain health literacy competencies or health literacy related communication skills training, as previously defined ^47, 48^ in order to be included. For the purpose of this research, the relational characteristic of HL is recognised and informs the choice of definition used which is that HL is ‘People’s ability to find, understand, appraise and communicate information to engage with the demands of different health contexts to promote health across the lifecourse’ as developed by Kwan (2006) ^10^. In this current study, qualified health professionals identified will not be limited by profession in which they work. It must be noted that this search is limited to adult patient populations as often foot screening begins in adulthood as diabetes is monitored ^58^. For the purpose of this study and the overarching project, health professional students will not be included in the population as the main focus is qualified health professionals working in diabetes care. Study selection will be based on the inclusion criteria provided in Table 2.

**Table 2.** Inclusion/Exclusion Criteria.

Inclusion criteria	Exclusion criteria
Population: Qualified health professionals.	Population: Healthcare students
Adult patient populations (>18 years old)	Patient population: Paediatric (<18 years old)
Intervention: HL competencies and HL related communication skills education containing competencies as previously defined ^47, 48^	Literature pre 1973
Study Methods: All research methodologies	Not in the English language
Limited to 1973- September 2021	
Settings: primary, secondary and tertiary care	

Similar to previous research, the selection of sources and evidence will take place over four steps ^59^:

Step 1: Initial retrieval of sources, which will be performed by one author.

Step 2: Title screening. Titles will be screened against the inclusion criteria and will be retained if they explicitly meet the inclusion criteria. This step will be performed by two blinded authors, whereby the third author will mediate if any disagreements arise.

Step 3: Abstract screening. Abstracts will be screened against the inclusion criteria and will be retained if they meet the inclusion criteria. This step will be performed by two blinded authors. Disagreements will be mediated by the third author through discussion.

Step 4: Full text review. Articles will be retained if compliant with inclusion criteria. This will be performed by two authors of the research team and cross-checked with the third if any complications arise. Numbers of articles included and excluded will be documented using the PRISMA-ScR standardised template ^53^.

### Stage 4: Charting the data

The extraction form will be collated based on the JBI template source of evidence details, characteristics and results extraction instrument ^52^, training programme evaluation methods ^60^ and insight from previous work ^61^. A data charting form will be developed drawing on categories, as agreed by the research team, such as: article details, demographics, intervention details, such as adult education approaches, HL domain implementation and evaluation methods. An excel spreadsheet will be used to chart the data.

### Stage 5: Collating, summarizing, and reporting of results

Data will be reported for each selected study within each category as agreed on in the previous stage. Findings will be presented in a table that outlines the research demographics as defined in Stage 4. Any subcategories of emerging themes will be identified depending on presenting data. Entries will be checked by all authors.

### Dissemination

The findings of this scoping review will be published in a peer-reviewed journal and made available on ARAN, an NUI Galway open access repository, subject to the open-access policies of the original publishers.

### Study status

Not yet initiated.

## Conclusions

Although some training programmes have been developed to address HL competencies and HL related communication skills ^37, 41, 42^, the extent and nature of programmes, needs identifying and collating to assess the potential of undertaking a full systematic review ^43^. This will inform future development of these complex interventions. Current educational health literacy interventions aimed at qualified health professionals need to be identified accordingly to collate the current evidence base and provide a comprehensive narrative pertaining to the characteristics, including their generic or any disease specific focus, methodologies and assessments used.

## Data availability

No data are associated with this article.
