# Supplementary material for: Health literacy education programmes developed for qualified health professionals: a scoping review protocol
Source: HRB Open Res. 2022 Jan 11;4:97. Originally published 2021 Sep 2. [Version 2] doi: 10.12688/hrbopenres.13386.2 (PMC8881692; doi:10.12688/hrbopenres.13386.2)
Supplement: Peer review history

# Peer review: Reviewer response for version 2

**DOI:** 10.21956/hrbopenres.14699.r31170

**Published:** 2022-02-25

**Authors:** Sarah Barry

**Affiliations:** 1 Centre for Health Policy and Management, Trinity College Dublin, Dublin, Ireland

The authors have responded to the points raised in my peer review report. The updates explain to a greater extent the theoretical underpinnings and operational elements of the original study protocol. Hopefully the queries raised have helped clarify the general frameworks that will drive the full programme of investigation envisaged. The full study can make a really useful contribution, especially for improved outcomes for diabetes patients.

Is the study design appropriate for the research question?

Yes

Is the rationale for, and objectives of, the study clearly described?

Partly

Are sufficient details of the methods provided to allow replication by others?

Yes

Are the datasets clearly presented in a useable and accessible format?

Not applicable

Reviewer Expertise:

integrated care, organisation science, policy implementation, health services

I confirm that I have read this submission and believe that I have an appropriate level of expertise to confirm that it is of an acceptable scientific standard.

# Peer review: Reviewer response for version 2

**DOI:** 10.21956/hrbopenres.14699.r31169

**Published:** 2022-01-24

**Authors:** Susie Sykes, Catherine Jenkins

**Affiliations:** 1 Institute of Health and Social Care, London South Bank University, London, UK

We are happy that the authors have addressed all of the points we raised in our review and that this protocol is now suitable for indexing.

 The authors may wish to note that one of the sources that they include in their protocol, OpenGrey, is being decommissioned and may not therefore be available to them.

Is the study design appropriate for the research question?

Yes

Is the rationale for, and objectives of, the study clearly described?

Partly

Are sufficient details of the methods provided to allow replication by others?

Partly

Are the datasets clearly presented in a useable and accessible format?

Yes

Reviewer Expertise:

Health literacy, critical health literacy, health literacy education for health professionals and students, scoping review design.

We confirm that we have read this submission and believe that we have an appropriate level of expertise to confirm that it is of an acceptable scientific standard.

# Peer review: Reviewer response for version 1

**DOI:** 10.21956/hrbopenres.14580.r30201

**Published:** 2021-10-15

**Authors:** Sarah Barry

**Affiliations:** 1 Centre for Health Policy and Management, Trinity College Dublin, Dublin, Ireland

The paper is a protocol for a scoping review of health literacy (HL) literature, with a particular focus on HL training for healthcare professionals working in all clinical settings, although some focus on professionals working with diabetes patients is suggested.

 The rationale for the scoping review is to map interventions (programmes, etc.) currently not evident in the literature as a starting point in considering the viability of a systemic review. A link between healthcare professional's HL competency and positive outcomes for patients is asserted. Subsequent study aims to inform better design and implementation of HL interventions based on a systematic review of the evidence scoped here.

 One of the important premises is that most HL interventions are functional in nature, e.g. better awareness of risks, services and adherence, whilst interactive and critical domains of HL are less evident. The methods for the review are comprehensively described and referenced in several stages, nonetheless, I find a few overall study design elements could be further developed. 

 In essence, I think the protocol and planned review would be strengthened if the general worldview underpinning the study were more evident. This means clarifying something like a relational framework for HL competencies and skills, contexts, etc., and how that functions in the patient-healthcare professional dyad (or system).

 Such a framework (encapsulating a worldview) would help for clarifying questions arising here such as, what would more critical HL add to this relationship and/or better outcomes for patients and/or professionals; or why focus on diabetes care and outcomes as a good case?

 Interactive and critical HL are linked to communication skills and greater relational competency, but other aspects could be explored including adult learning approaches. I think more detail on critical HL is needed, especially given the authors include all clinical settings in their inclusion criteria. Without knowing this literature in-depth I imagine critical HL would have to consider some of the institutionalised and systemic aspects of professional-patient interactions and outcomes given a relationship-based framework. I guess there is potential here for clinicians and patients together to become better system navigators.

 A qualification of the secondary research question No. 2 (What are the characteristics of each education programme?) could suggest categorising programmes as 'functional', 'interactive' or 'critical' as a way to better understand the nature of these differences or the outcomes they generate.

 A second element of the study design somewhat missing is evidence on HL among healthcare professionals. HL among diabetes patients is reported here, but how this evidence links to HL outcomes among professionals is not developed sufficiently.

 Given the focus here is on health professionals and the HL interventions available to them, or indeed accessed by them - I am missing the literature that says something about this population in particular - even if scant and shows some sort of context-mechanism-outcome pattern. Some examples of positive outcomes from HL for healthcare professionals might include leadership skills development, skills in policy advocacy, or access to career development opportunities.

 As per above, some more descriptions of why it is a good idea to focus on the relationship between diabetes patients and diabetes care (providing) professionals would add to the overall rationale. I imagine this can be easily asserted given the size of the population in question.

 I thought the use of population, concept and context (PCC) as a frame for the review is a useful focusing plan. I'm not sure how this is a mnemonic (as stated), or how it functions to operationalise the study. I would expect a brief outline of the plan to report findings that corresponds to the PCC approach, and maybe explaining what this adds.

 I also wondered why the authors are not planning a 'stage 7' stakeholder engagement as part of the scoping review - especially given their sub-question on implementation. The reason may be lack of funding, time, etc. If this is the case it would be good to say so.

 Overall, the protocol positively outlines the rationale, design and next steps for studying HL among healthcare professionals as an addition to both the literature and practice. Mapping current interventions is a positive contribution that will build development of better interventions. The protocol would gain from more exploration of its ontological approach - I think this is implied but not fully stated or its implications drawn out.

Is the study design appropriate for the research question?

Yes

Is the rationale for, and objectives of, the study clearly described?

Partly

Are sufficient details of the methods provided to allow replication by others?

Yes

Are the datasets clearly presented in a useable and accessible format?

Not applicable

Reviewer Expertise:

integrated care, organisation science, policy implementation, health services

I confirm that I have read this submission and believe that I have an appropriate level of expertise to confirm that it is of an acceptable scientific standard, however I have significant reservations, as outlined above.

# Response

**Published:** 2021-12-15

**Authors:** Lauren Connell

**Affiliations:** National University of Ireland, Galway, Ireland

Dear Dr Sarah Barry,

 Thank you for reviewing our protocol submission, and for your constructive feedback. As a result, revisions have been made and are individually addressed below, please see reviewer’s comments in bold. The revisions suggested from your comments certainly enhance the overall protocol.

**The paper is a protocol for a scoping review of health literacy (HL) literature, with a particular focus on HL training for healthcare professionals working in all clinical settings, although some focus on professionals working with diabetes patients is suggested. The rationale for the scoping review is to map interventions (programmes, etc.) currently not evident in the literature as a starting point in considering the viability of a systemic review. A link between healthcare professional's HL competency and positive outcomes for patients is asserted. Subsequent study aims to inform better design and implementation of HL interventions based on a systematic review of the evidence scoped here. One of the important premises is that most HL interventions are functional in nature, e.g. better awareness of risks, services and adherence, whilst interactive and critical domains of HL are less evident. The methods for the review are comprehensively described and referenced in several stages, nonetheless, I find a few overall study design elements could be further developed.**

 Thank you for your positive comments, please see responses below.

**In essence, I think the protocol and planned review would be strengthened if the general worldview underpinning the study were more evident. This means clarifying something like a relational framework for HL competencies and skills, contexts, etc., and how that functions in the patient-healthcare professional dyad (or system).**

 This study is part of a larger project focussed on diabetic foot disease (DFD) prevention, and this project aims to improve interactive health literacy (HL) from a communicative point of view. A relational concept of health literacy will be used (1), focusing on an organisational health literacy (OHL) approach which makes health services easier for patients and their families to access, navigate and engage with so that they can make informed decisions and take informed actions for their health (2). By adopting this approach, increasing HL competencies and communication has the potential to strengthen the patient-healthcare professional dyad. Please see amended protocol introduction that introduces OHL and the relational concept of HL. Reference to this is now included in the update protocol. See introduction paragraph 3.

**Such a framework (encapsulating a worldview) would help for clarifying questions arising here such as, what would more critical HL add to this relationship and/or better outcomes for patients and/or professionals; or why focus on diabetes care and outcomes as a good case?**

 The concept of OHL is an important one that helps us determine the relevance and understanding of where interactive HL comes into the overall study. Predominately the literature focuses on functional HL, and the literacy proficiency needed to navigate the health system. In the overall PhD project the objective is to target interactive HL by addressing patient-practitioner communication and the therapeutic relationship. This will be achieved by developing an education programme to improve the interactive domain of HL, and introduce a shift in understanding for professionals whereby HL is often an under/overestimated concept when it comes to patient interaction. Attaining critical HL is the ultimate goal in creating an accessible and inclusive health system, where individuals can evaluate and critique relevant health information. Therefore, by attaining critical HL at a community level, individuals have the potential to use the patient-professional consultation to its full capacity in promoting health creating a cultural shift.

 The worldview is addressed within the protocol introduction (paragraph 3) where the reason for the focus on diabetes is included. Reference to this is now included in the updated protocol, see introduction paragraphs 5,6 and 7.

**Interactive and critical HL are linked to communication skills and greater relational competency, but other aspects could be explored including adult learning approaches.**

 Adult learning approaches and methodologies will be reported in the results when charting the retrieved data. It is intended to explore this in the next stages of intervention development, where experiential learning (3) will be explored in further detail. Reference to the inclusion of adult learning approaches and methodologies is now explicitly included in the protocol methodology stage 4: charting the data.

**I think more detail on critical HL is needed, especially given the authors include all clinical settings in their inclusion criteria. Without knowing this literature in-depth I imagine critical HL would have to consider some of the institutionalised and systemic aspects of professional-patient interactions and outcomes given a relationship-based framework. I guess there is potential here for clinicians and patients together to become better system navigators.**

 This current study is focused on the interactive aspects of HL and the patient practitioner interaction, therefore the chosen settings, primary, secondary and tertiary, aim to capture all qualified health professionals where patient communication is needed. However, it must be noted that critical HL is attributed to having advanced personal and social skills enabling one to access, manage, assess the credibility, understand and critically appraise information on health related issues (4). Critical HL is seen to arise from the relationship between individuals and services, being able to navigate and advocate for themselves in the healthcare setting. Reference to this is now included in the updated protocol, see methodology paragraphs 1 and 2.

**A qualification of the secondary research question No. 2 (What are the characteristics of each education programme?) could suggest categorising programmes as 'functional', 'interactive' or 'critical' as a way to better understand the nature of these differences or the outcomes they generate.**

 Thank you for this suggestion. It is anticipated that such categories will be recorded, as it will demonstrate meaningful information when carried out in charting the results and is explicitly included in Stage 4: Charting the data.

**A second element of the study design somewhat missing is evidence on HL among healthcare professionals. HL among diabetes patients is reported here, but how this evidence links to HL outcomes among professionals is not developed sufficiently.**

 The need for health professionals HL education, to improve patient health outcomes, has been identified (5), is supported by research literature (5-7) and is recognised in policy development in European countries (8). Educating health professionals has the ability to make an impact in reducing health inequalities in populations at the highest risk of limited HL levels, particularly within diabetes. It is suggested that when HL is considered in isolation it is associated with greater diabetes self-efficacy (9-11), where greater self-efficacy is associated with lower glycaemic levels.  Inadequate HL has been shown to be an independent predictor of poor glycaemic control, being associated with a lower likelihood of achieving tight control (12). Similarly, HL was associated with a higher prevalence of retinopathy and other self-reported complications of diabetes (12). This is now included in the protocol Introduction paragraph 7.

**Given the focus here is on health professionals and the HL interventions available to them, or indeed accessed by them - I am missing the literature that says something about this population in particular - even if scant and shows some sort of context-mechanism-outcome pattern. Some examples of positive outcomes from HL for healthcare professionals might include leadership skills development, skills in policy advocacy, or access to career development opportunities.**

 In terms of  professional outcomes it is intended that if an organisation is health literate that individuals working within will display OHL attributes such as leadership, HL integration into planning, community engagement, use of HL strategies in communication, designing accessible resources and clear communication (13)

**As per above, some more descriptions of why it is a good idea to focus on the relationship between diabetes patients and diabetes care (providing) professionals would add to the overall rationale. I imagine this can be easily asserted given the size of the population in question.**

 The evidence base for interventions to prevent diabetic foot disease is lacking. Therefore, to address this unmet and critical need, this Collaborative Doctoral award programme of research is focusing on primary and secondary prevention of DFD and novel treatment approaches to improve patient outcomes for those with DFD. The overall goal of DFD PRIMO is to train a multidisciplinary cohort of health care professionals to doctoral level in order to increase internationally competitive DFD research activity in Ireland, to provide a strong evidence-base for prevention and treatment provision decisions and improved patient outcomes.

**I thought the use of population, concept and context (PCC) as a frame for the review is a useful focusing plan. I'm not sure how this is a mnemonic (as stated), or how it functions to operationalise the study. I would expect a brief outline of the plan to report findings that corresponds to the PCC approach, and maybe explaining what this adds.**

 The PCC (Population (or participants)/Concept/Context) is a framework recommended by the Joanna Briggs Institute. It enables one to identify the main concepts in the primary review question to allow for added structure within development of the study’s aims and criteria. This is now addressed in Methodology paragraph 2. However, the PCC framework is designed to be utilised in creating the review title and the planning of the review, therefore it will not be used as a tool to report findings.

**I also wondered why the authors are not planning a 'stage 7' stakeholder engagement as part of the scoping review - especially given their sub-question on implementation. The reason may be lack of funding, time, etc. If this is the case it would be good to say so.**

 The optional stage which comprises stakeholder consultation will not be adopted in the context of this current study. However, this research is the first stage to a three stage project which aims to incorporate stakeholder engagement informed by and using data collated from this review. This is noted within the updated protocol under Methodology paragraph 3.

**Overall, the protocol positively outlines the rationale, design and next steps for studying HL among healthcare professionals as an addition to both the literature and practice. Mapping current interventions is a positive contribution that will build development of better interventions. The protocol would gain from more exploration of its ontological approach - I think this is implied but not fully stated or its implications drawn out.**

 In terms of ontological approach, the UK Medical Research Council (MRC) framework on developing and evaluating complex interventions (14) will be used to guide this research programme, whereby the four stage process will be used to develop a complex intervention informed by a gap analysis (scoping review), expert consultation and review. This framework recommends a phased development process, which is indeed the case for this research. It allows a continuum of increasing evidence in order to assist with intervention development (14). This approach uses systems theory which is a foundation for OHL, in order to structure intervention development. HL is a relational concept whereby focus is on individual interaction with services and systems, from an OHL point of view. As the focus is on OHL, it is anticipated that a more health literate organisation will result in reduced barriers for individuals accessing and utilising healthcare.

**Reference List:**

1. Zumbo B, Kelly K, Begoray D, Kazanjian A, Mullet J, Hayes M. The development and validation of measures of “Health Literacy” in different populations. UBC Institute of Health Promotion Research and UVic Community Health Promotion Research: University of British Columbia. 2006.
2. Brega AG, Hamer MK, Albright K, Brach C, Saliba D, Abbey D, *et al.* Organizational health literacy: Quality improvement measures with expert consensus. HLRP: Health Literacy Research and Practice. 2019;3(2):e127-e46.
3. Kolb DA. Experience as the source of learning and development. Upper Sadle River: Prentice Hall. 1984.
4. Sykes S, Wills J, Rowlands G, Popple K. Understanding critical health literacy: a concept analysis. BMC public health. 2013;13(1):1-10.
5. Nutbeam D, Lloyd JE. Understanding and Responding to Health Literacy as a Social Determinant of Health. Annu Rev Public Health. 2021;42:159-73.
6. Mackert M, Ball J, Lopez N. Health literacy awareness training for healthcare workers: Improving knowledge and intentions to use clear communication techniques. Patient Education and Counseling. 2011;85(3):e225-e8.
7. Saunders C, Palesy D, Lewis J. Systematic Review and Conceptual Framework for Health Literacy Training in Health Professions Education. Health Professions Education. 2019;5(1):13-29.
8. Okan O, Bauer U, Levin-Zamir D, Pinheiro P, Sørensen K. International Handbook of Health Literacy: Research, practice and policy across the lifespan: Policy Press; 2019.
9. Osborn CY, Cavanaugh K, Wallston KA, Rothman RL. Self-efficacy links health literacy and numeracy to glycemic control. Journal of health communication. 2010;15(S2):146-58.
10. Cavanaugh K, Huizinga MM, Wallston KA, Gebretsadik T, Shintani A, Davis D, *et al.* Association of numeracy and diabetes control. Annals of internal medicine. 2008;148(10):737-46.
11. Bohanny W, Wu SFV, Liu CY, Yeh SH, Tsay SL, Wang TJ. Health literacy, self‐efficacy, and self‐care behaviors in patients with type 2 diabetes mellitus. Journal of the American Association of Nurse Practitioners. 2013;25(9):495-502.
12. Schillinger D, Grumbach K, Piette J, Wang F, Osmond D, Daher C, *et al.* Association of health literacy with diabetes outcomes. Jama. 2002;288(4):475-82.
13. Brach C, Keller D, Hernandez LM, Baur C, Parker R, Dreyer B, *et al.* Ten attributes of health literate health care organizations. NAM Perspectives. 2012.
14. Skivington K, Matthews L, Simpson SA, Craig P, Baird J, Blazeby JM, *et al.* A new framework for developing and evaluating complex interventions: update of Medical Research Council guidance. bmj. 2021;374.

# Peer review: Reviewer response for version 1

**DOI:** 10.21956/hrbopenres.14580.r30205

**Published:** 2021-09-16

**Authors:** Susie Sykes, Catherine Jenkins

**Affiliations:** 1 Institute of Health and Social Care, London South Bank University, London, UK

Thank you for the opportunity to review this protocol which we feel forms the basis of an important and useful scoping review. This protocol for a scoping review addresses an important area of inquiry of an emerging area of research that addresses a practice-based problem. The review is well-planned and largely methodologically sound but issues of replicability could be enhanced. This is discussed below. It is aligned with the PRISMA ScR guidance and has benefitted from the inclusion of a librarian to design the search strategy. We agree that a scoping review is the most appropriate review type to explore the research objectives outlined here and to assess the need for undertaking a systematic review in the future. The inclusion of grey literature will enrich the review. Incorporating grey literature coverage also provides further justification for the choice of review type.

 The authors provide a clear rationale for conducting a scoping review to address their research objectives, which are clearly-defined.

 Some points that the authors may wish to consider:

- While the rationale for the study clearly states the prevalence and implications of low health literacy amongst patients and the public, there is a slight leap between this and the specific problem the scoping review seeks to address of health professional training/education. While it is perhaps implicit, a clear delineation of exactly what is included in your understanding of health literacy education for professionals would be helpful. Is it to improve the health literacy of professionals themselves, their understanding of how to respond to low and varied health literacy levels of patients or how to create a health literate environment. We think that there is value in drawing on some of the literature around health literacy as a relational concept that explores the relationship between the health literacy competencies of individuals and the demands of the environment.
- The research questions are clear and helpful but a secondary question around diabetes is introduced and the rationale for this over other types of subject-specific health literacies is not clearly made.
- We feel there is a chance that a bias could be introduced at the study selection stage stemming from the intervention inclusion criteria (and this links with our first point). The health literacy competencies have not been clearly articulated and are open to interpretation. There also appears to be a conflation between health literacy education and communication skills education and this needs clarity. They are not synonymous. It is not clear to us exactly what you are including in your intervention criteria and why. This undermines the replicability of this study.
- It is not clear why the study excludes health care students or pediatric patient populations.
- Is the HSE health research repository distinct enough from Lenus to be included as a separate grey literature source? Perhaps Carrot2, OpenGrey or Google Scholar UK (in an incognito browser) could be substituted (providing a more balanced mix: two Irish and two international grey literature sources).
- There is a pre-1973 source related to HL which you may wish to take into account in your date range, although it does not focus on education: Dixon, J.P. (1959). The community responsibility for medical care. Am J Public Health 49, 76–81. https://doi.org/10.2105/AJPH.49.1.76. ^1^
- Will your end-date of 2021 in the search strategy capture preprints and reviews-in-progress in e.g. PROSPERO?
- The PCC stipulates a clinical setting, but the inclusion criteria stipulate all settings. Is this a discrepancy?

Is the study design appropriate for the research question?

Yes

Is the rationale for, and objectives of, the study clearly described?

Partly

Are sufficient details of the methods provided to allow replication by others?

Partly

Are the datasets clearly presented in a useable and accessible format?

Yes

Reviewer Expertise:

Health literacy, critical health literacy, health literacy education for health professionals and students, scoping review design.

We confirm that we have read this submission and believe that we have an appropriate level of expertise to confirm that it is of an acceptable scientific standard, however we have significant reservations, as outlined above.

## References

[1] : The community responsibility for medical care. *Am J Public Health Nations Health* .1959;49(1) : 10.2105/ajph.49.1.76 76-81 10.2105/ajph.49.1.76 13617537PMC1372646

# Response

**Published:** 2021-12-15

**Authors:** Lauren Connell

**Affiliations:** National University of Ireland, Galway, Ireland

Dear Dr Susie Sykes,

 Thank you for reviewing our protocol submission, and for your very constructive feedback. As a result, revisions have been made and are individually addressed below, please see reviewer’s comments in bold. The revisions suggested from your comments certainly enhance the overall protocol.

**Thank you for the opportunity to review this protocol which we feel forms the basis of an important and useful scoping review. This protocol for a scoping review addresses an important area of inquiry of an emerging area of research that addresses a practice-based problem. The review is well-planned and largely methodologically sound but issues of replicability could be enhanced. This is discussed below. It is aligned with the PRISMA ScR guidance and has benefitted from the inclusion of a librarian to design the search strategy. We agree that a scoping review is the most appropriate review type to explore the research objectives outlined here and to assess the need for undertaking a systematic review in the future. The inclusion of grey literature will enrich the review. Incorporating grey literature coverage also provides further justification for the choice of review type.**

 Thank you for your positive comments. Please see responses below.

**While the rationale for the study clearly states the prevalence and implications of low health literacy amongst patients and the public, there is a slight leap between this and the specific problem the scoping review seeks to address of health professional training/education. While it is perhaps implicit, a clear delineation of exactly what is included in your understanding of health literacy education for professionals would be helpful. Is it to improve the health literacy of professionals themselves, their understanding of how to respond to low and varied health literacy levels of patients or how to create a health literate environment.**

 Thank you for this important observation. This has been clarified within the updated manuscript where a more explicit connection has been made between the problem statement and the background of health literacy.

 In the context of the development of organisational health literacy, health literacy education aims to address areas that health professionals can be trained in order to appropriately respond to and address limited and variable levels of health literacy in the patient population, this can be achieved by using techniques to encourage adequate HL, such as Teach-Back and avoiding medical jargon, which confirm understanding (1), whilst designing health literate reading materials to improve comprehensibility (2). Health professionals have an impact on overall organisational health literacy, in confirming understanding and interpersonal communication (1, 3). Therefore, by targeting health professionals there will be an organisational impact. In terms of  professional outcomes it is intended that if an organisation is health literate that individuals working within will display OHL attributes such as leadership, HL integration into planning, community engagement, use of HL strategies in communication, designing accessible resources and clear communication (4).  

**The research questions are clear and helpful but a secondary question around diabetes is introduced and the rationale for this over other types of subject-specific health literacies is not clearly made.**

 Thank you for this observation, focus on diabetes is something that has been addressed in the manuscript. This particular review is a component of a larger funded research project comprising multiple doctoral students with multiple projects that focuses on diabetic foot disease and its prevention. Therefore, it was decided to scope the literature for any relevant health literacy education programmes that have been implemented within diabetes care. Sequentially, a prototype health literacy intervention will be developed and increasing focus will be placed on professionals working in the diabetes multidisciplinary team as the project progresses. Where the scoping review is situated in the study as a whole and the context of the larger research programme are now included in the manuscript.

****

**We feel there is a chance that a bias could be introduced at the study selection stage stemming from the intervention inclusion criteria (and this links with our first point). The health literacy competencies have not been clearly articulated and are open to interpretation. There also appears to be a conflation between health literacy education and communication skills education and this needs clarity. They are not synonymous. It is not clear to us exactly what you are including in your intervention criteria and why. This undermines the replicability of this study.**

 Thank you for this comment, this is a clear limitation of the protocol. The health literacy competencies have been defined in line with previous research (5, 6) whereby competencies have been established and are articulated clearly. Similarly, key attributes of a health literate organisation  have been established (4).

 Communication skills education is recognised to be a component of HL education from the point of view of ‘oral exchange’ and interpersonal communication between the HP and the patient. They are not seen as synonymous but they are interlinked, in particular when the aim of communication skills education is to develop competencies that promote health literacy training of health professionals (7). In teaching HPs HL techniques the goal is to enhance the patient’s understanding, not to change, explain or understand behaviour but to encourage the absorption of health information in order that the patient can make informed decisions and take informed actions.

**It is not clear why the study excludes health care students or pediatric patient populations.**

 Because this study is a part of a larger project, it was decided to look at qualified health professional education, as it is emerging; the author recommends a separate review to explore health literacy education in the health professions’ student curricula. Patient populations that encompass those most at risk of diabetic disease i.e. adult patient populations, as often diabetic foot screening begins in adulthood and continues to be monitored throughout adulthood (8). The Collaborative Doctoral Award (CDA) in which this project is a part is focused on diabetic foot disease. The reason for excluding healthcare students and paediatric populations is now included in the protocol within the methodology section Stage 3: Study selection.

****

**Is the HSE health research repository distinct enough from Lenus to be included as a separate grey literature source? Perhaps Carrot2, OpenGrey or Google Scholar UK (in an incognito browser) could be substituted (providing a more balanced mix: two Irish and two international grey literature sources).**

 Thank you for this comment, amendments have been made to remove the HSE health research repository, and include a more balanced mix of grey literature sources as suggested.

****

**There is a pre-1973 source related to HL which you may wish to take into account in your date range, although it does not focus on education: Dixon, J.P. (1959). The community responsibility for medical care. Am J Public Health 49, 76–81.** **https://doi.org/10.2105/AJPH.49.1.76.1**

 Thank you for this, it has been insightful to read. However, it does not meet the study’s inclusion criteria or address health literacy education in health professionals.

****

**Will your end-date of 2021 in the search strategy capture preprints and reviews-in-progress in e.g. PROSPERO?**

 September 2021 was used and there is no exclusion criteria regarding study type therefore it is possible that the search strategy will capture those studies. Although, the study aims to capture characteristics of education programmes and feasibility outcomes, which may not be accessible when including a review in progress.

**The PCC stipulates a clinical setting, but the inclusion criteria stipulate all settings. Is this a discrepancy?**

 Settings will include primary, secondary and tertiary care settings. The protocol has been amended to reflect this change.

**Reference List:**

1. Brach C. The journey to become a health literate organization: a snapshot of health system improvement. Studies in health technology and informatics. 2017;240:203.
2. DeWalt DA, Broucksou KA, Hawk V, Brach C, Hink A, Rudd R, *et al. *Developing and testing the health literacy universal precautions toolkit. Nursing outlook. 2011;59(2):85-94.
3. Brach C, Harris LM. Healthy People 2030 health literacy definition tells organizations: make information and services easy to find, understand, and use. Journal of General Internal Medicine. 2021;36(4):1084-5.
4. Brach C, Keller D, Hernandez LM, Baur C, Parker R, Dreyer B, *et al.* Ten attributes of health literate health care organizations. NAM Perspectives. 2012.
5. Karuranga S, Sørensen K, Coleman C, Mahmud AJ. Health literacy competencies for European health care personnel. HLRP: Health Literacy Research and Practice. 2017;1(4):e247-e56.
6. Coleman C, Kurtz-Rossi S, McKinney J, Pleasant A, Rootman I, Shohet L. The Calgary charter on health literacy: Rationale and core principles for the development of health literacy curricula. The Center for Literacy of Quebec. 2008.
7. Nutbeam D, Lloyd JE. Understanding and Responding to Health Literacy as a Social Determinant of Health. Annu Rev Public Health. 2021;42:159-73.
8. Hurley L, Kelly L, Garrow AP, Glynn LG, McIntosh C, Alvarez-Iglesias A, *et al.* A prospective study of risk factors for foot ulceration: The West of Ireland Diabetes Foot Study. QJM: An International Journal of Medicine. 2013;106(12):1103-10.
